# Phase-Sensitive Fluorescence Image Correlation Spectroscopy

**DOI:** 10.3390/ijms252011165

**Published:** 2024-10-17

**Authors:** Andrew H. A. Clayton

**Affiliations:** Optical Sciences Centre, Department of Physics and Astronomy, School of Science, Computing and Engineering Technologies, Swinburne University of Technology, Hawthorn, Melbourne, VIC 3122, Australia; aclayton@swin.edu.au

**Keywords:** FLIM, image correlation spectroscopy, membrane probe

## Abstract

Fluorescence lifetime imaging microscopy is sensitive to molecular interactions and environments. In homo-dyne frequency-domain fluorescence lifetime imaging microscopy, images of fluorescence objects are acquired at different phase settings of the detector. The detected intensity as a function of detector phase is a sinusoidal function that is sensitive to the lifetime of the fluorescent species. In this paper, the theory of phase-sensitive fluorescence image correlation spectroscopy is described. In this version of lifetime imaging, image correlation spectroscopy analysis (i.e., spatial autocorrelation) is applied to successive fluorescence images acquired at different phase settings of the detector. Simulations of different types of lifetime distributions reveal that the phase-dependent density of fluorescent objects is dependent on the heterogeneity of lifetimes present in the objects. We provide an example of this analysis workflow to a cervical cancer cell stained with a fluorescent membrane probe.

## 1. Introduction

Fluorescence lifetime imaging microscopy (FLIM) is a powerful approach to examine decay rates of excited states in an image [1,2,3]. Its utility derives from the exquisite environmental sensitivity of fluorescence lifetimes (i.e., polarity, pH, refractive index, FRET, and metabolic index), enabling the spatial mapping of molecular interactions and environments. In principle, lifetime imaging delivers pixel-by-pixel resolution of fluorescence lifetimes with the goal of providing a map or a 2D image containing contrast. However, in practice, noise can broaden lifetime d, giving the impression of lifetime heterogeneity. For example, detected lifetimes in an image may vary due to true changes in lifetime in a complex structure such as a living cell, or alternatively due to pixel-by-pixel noise in an otherwise homogenous sample [4]. Another consideration is that real fluorescent objects produce fluorescence that is smeared in image space by the optical transfer function of the microscope, which, depending on the characteristics of the object, microscope, and detection system, may cover several pixels. Translational motion of particles in an image can also cause apparent lifetime broadening, since the ensuing concentration fluctuations will add noise to the lifetime image determination [5]. Conversely, a high density of objects may cause the averaging of lifetime information per pixel or voxel. Furthermore, images derived from fluorescence lifetimes can sometimes have little discernible contrast because the variations in lifetime from different environments are occurring at the molecular level, and are partially averaged or randomized by processes foreshadowed above.

Image correlation spectroscopy (ICS) [6,7,8,9,10,11,12,13] is a particularly advantageous method for the analysis of fluorescence images (which otherwise appear diffuse and to contain random noise) due to spatially correlated fluorescence fluctuations. The key in terms of noise to image correlation spectroscopy is that pixel-by-pixel noise contributes to the zero-lag autocorrelation, while true fluorescence spans several pixels (the autocorrelation for molecular-sized fluorescence objects will decay as a Gaussian function with a decay length of the order of the point-spread function of the microscope or of about 250 nm). Additionally, image correlation spectroscopy provides estimates of the average particle densities and relative brightnesses of fluorescence species in a fluorescence image.

There are several potential ways to combine ICS with FLIM. Spatial autocorrelation analysis of FLIM images has been recently employed by Coppey-Moisan’s group to look at the spatial scale of lifetime fluctuations in chromatin in the nucleus of cells [14]. In that work, the authors used the decay of the fluorescence lifetime fluctuation autocorrelation function with spatial lag to infer the relevant spatial scales involved. Our lab [15] combined FLIM with ICS to extract cluster densities and the relative brightness of FRET and non-FRET states to examine the link between receptor clustering and adaptor binding. The approach uses a two-state FLIM model to determine lifetimes of FRET and non-FRET states, and then uses this information to extract fluorescence images of FRET and non-FRET states, which are subsequently analyzed by conventional ICS. The approach can be applied to systems containing two different lifetimes and the information from this analysis is the cluster density of different lifetime species and the relative brightness. Here, we propose yet another approach. We retain the idea of using image correlation on fluorescence images, but use the inherent phase-sensitive nature of our FLIM system to vary the contribution of different lifetime states in the image by adjusting the phase of the detector. To be explicit, in our frequency-domain homo-dyne microscope, the excitation intensity and detector gain are driven at the same frequency, but can be shifted in phase to generate phase-sensitive fluorescence images. Depending on the species lifetime, different lifetime species can be in-phase or out-of-phase with the detector and so enhanced or depressed relative to other lifetime states. Accordingly, we anticipate that for an image with true lifetime heterogeneity, the apparent density of particles, as defined by image correlation, will be modulated with phase of the detector.

The paper is organized as follows. In the Theory Section, we will outline the principles of phase-sensitive fluorescence and ICS and derive the theoretical expressions for psf–ICS. In the Results Section, we will show plots for idealized models of lifetime heterogeneity including two-state, Gaussian distribution and Lorentzian distribution. We will show that psf–ICS is sensitive to the heterogeneity in the lifetimes in an image. As a pedagogical example, we will demonstrate an analysis workflow of FLIM and psf–ICS with data collected from a cell labelled with a membrane dye.

## 2. Theory

### 2.1. FLIM and Phase-Sensitive Fluorescence

In frequency-domain FLIM, the sample is excited with a sinusoidally modulated light.

(1) $$I_{exc}\left(t\right)=1+m_{exc}\sin\omega t$$

where *m_exc_* is the modulation of the exciting light and *ω* is the circular frequency (in rad/s).

Owing to the finite lifetime of the excited state, the emission from the sample will be modulated at the same frequency as the excitation light, but demodulated (by a factor *m_em_*) and phase-shifted (by *φ_em_* radians) relative to the excitation:

(2) $$I_{em}\left(t\right)=1+m_{exc}m_{em}\sin\left(\omega t-\varphi_{em}\right)$$

If the emission is from a single exponential decaying fluorescence with lifetime *τ*_1_, then this lifetime is related to the measured phase and modulation of the detected emission via Equations (3) and (4):

(3) $$\tau_{1}=(\frac{1}{\omega })\tan\varphi_{em}$$

(4) $$M_{em}=\frac{1}{\sqrt{1+{\left(\omega \tau_{1}\right)}^{2}}}$$

In phase-sensitive detection, the detected intensity is proportional (proportionality constant, *k*) to the steady-state intensity (DC), the modulation of the emitted state (*m_em_*) and the cosine of the phase difference between the emission (*φ*) and the detector (*φ_D_*):

(5) $$I_{psf}\left(\varphi_{D}\right)=k(DC+{DCm}_{em}\cos(\varphi_{D}-\varphi_{em})$$

If there is more than one lifetime species Equation (5) becomes Equation (6):

(6) $$I_{psf}\left(\varphi_{D}\right)=k\sum_{i}\left({DC}_{i}+{{DC}_{i}m}_{i}\cos(\varphi_{D}-\varphi_{i}\right)$$

### 2.2. Image Correlation Spectroscopy

In conventional ICS (8), the intensity fluctuation spatial autocorrelation function is computed from an image I(x,y) using two-dimensional fast Fourier transform algorithm. The expression for the intensity fluctuation spatial autocorrelation function is

(7) $$g_{11}\left(\varepsilon ,\rho \right)=\frac{F^{-1}\left[F(I\left(x,y\right))*F^{*}(I\left(x,y\right))\right]}{{\left(\left|I\left(x,y\right)\right|\right)}^{2}}-1$$

where *F* represents the Fourier transform; *F*^−1^ the inverse Fourier transform; F* its complex conjugate; and the values *ε* and *ρ* are spatial lag variables.

The autocorrelation at zero lag, g_11_(0,0), provides the measurement of the inverse mean number of particles per beam area and is obtained by fitting the spatial autocorrelation function to a two-dimensional Gaussian function:

(8) $$g_{11}\left(\varepsilon ,\rho \right)=g_{11}\left(0,0\right)exp\left[\frac{\varepsilon^{2}+\rho^{2}}{\omega^{2}}\right]+g_{\infty }$$

In Equation (8), g_∞_ is an offset to account for long-range spatial correlations and ω is the full width at half maximum of the spatial autocorrelation function. For the remainder of the paper, we will refer to the autocorrelation at zero lag as simply g(0).

It is convenient to use the quantities’ cluster density (*CD*) and average brightness (B) as more intuitive measures of the particle distribution in an image. The cluster density can be readily obtained from the amplitude and width of the autocorrelation function, i.e.,

(9) $$CD={\left(g\left(0\right)\pi \omega^{2}\right)}^{-1}$$

where *CD* is the cluster density in units of number of particles per beam area. The beam area is given by *πω*^2^. The average brightness, B, is just the average image intensity divided by the cluster density. For the remainder of the paper will report the inverse of the autocorrelation amplitude at zero lag as the *CD* in the units of particles per beam area without specifying the actual beam area itself. This is justified on the basis that changing the phase of the detector will not change the size of the optical transfer function of the microscope.

It is important to note that the *CD* and B are well defined for a homogenous brightness distribution, i.e., all particles have the same brightness. When particles do not have the same brightness, *CD* and B are apparent quantities.

For a distribution of particles with cluster density *CD_j_* and brightness *B_j_*_,_ the apparent cluster density of the population, *CD_app_*, is given by the expression in Equation (10):

(10) $${CD}_{app}=\frac{{(\sum {CD}_{j}B_{j})}^{2}}{(\sum {CD}_{j}{B_{j}}^{2})}$$

It is clear from Equation (10) that if *j* = 1, then *CD_app_* = *CD*, as required.

### 2.3. Phase-Sensitive Fluorescence Image Correlation Spectroscopy

Having defined phase-sensitive fluorescence detection and image correlation spectroscopy, we now wish to combine the two approaches, which we will refer to as phase-sensitive fluorescence image correlation spectroscopy. We will consider a number of scenarios and derive the relevant expressions for those cases.

Homogenous lifetime image. Consider an (unmodulated) image containing fluorescence species dispersed at a cluster density *CD*. The average intensity of the image is <I> and therefore the brightness of each fluorescence species is <I>/*CD*. For the homogenous lifetime case, the lifetime of all species is given by *τ*.

We will now consider ICS analysis on each of the phase-sensitive images obtained using a phase-sensitive detector. The ICS analysis will generate an apparent cluster density at each phase setting, *φ_D_*, of the detector, which is denoted by *CD*(*φ_D_*). The cluster density as a function of detector phase is given by the following expression:

(11) $$CD\left(\varphi_{D}\right)=\frac{{\left(CDI_{psf}\left(\varphi_{D}\right)\right)}^{2}}{CD{I_{psf}\left(\varphi_{D}\right)}^{2}}=CD$$

Thus, for a homogenous lifetime image, the apparent cluster density should be independent of detector phase, but the average image intensity will be modulated according to Equation (5). Note that Equation (11) still holds even if the fluorescence decay is non-single exponential. The only requirement for Equation (11) to be valid is that the excited state decay behavior is invariant to spatial location (in the image).

Inhomogenous lifetime image (two spatially distinct lifetime species). We now consider the unmodulated image from two species with cluster densities *CD*_1_ and *CD*_2_ and average brightness contributions *B*_1_ and *B*_2_.

From Equation (10), the cluster density (for unmodulated excitation) will be given by

(12) $${CD}_{tot}=\frac{{{(CD}_{1}B_{1}+{CD}_{2}B_{2})}^{2}}{{(CD}_{1}{B_{1}}^{2}+{CD}_{2}{B_{2}}^{2})}$$

As expected, the apparent cluster density for a mixture of two different species is a weighted average of the two and there is no way of knowing the presence of the particle heterogeneity from the *CD_tot_* alone.

The apparent total cluster density as a function of detector phase angle for the two-species case is given by

(13) $$\begin{array}{ll} {CD\left(\varphi_{D}\right)}_{tot}= & \frac{{\left({CD}_{1}I_{psf1}\left(\varphi_{D}\right)+{CD}_{2}I_{psf2}\left(\varphi_{D}\right)\right)}^{2}}{\left({CD}_{1}{I_{psf1}\left(\varphi_{D}\right)}^{2}+{CD}_{2}{I_{psf2}\left(\varphi_{D}\right)}^{2}\right)}\\ & \;=\frac{{\left({CD}_{1}(B_{1}+B_{1}m_{1}\cos(\varphi_{D}-\varphi_{1}))+{CD}_{2}(B_{2}+B_{2}m_{2}\cos\left(\varphi_{D}-\varphi_{2}\right)\right)}^{2}}{\left({CD}_{1}(B_{1}+B_{1}m_{1}\cos\left(\varphi_{D}-\varphi_{1}\right){)}^{2}+{CD}_{2}{(B_{2}+B_{2}m_{2}\cos\left(\varphi_{D}-\varphi_{2}\right))}^{2}\right)} \end{array}$$

From Equation (13), if *φ*_1_ = *φ*_2_ then the apparent *CD* will be independent of the detector phase. However, if *φ*_1_ ≠ *φ*_2_ then the apparent *CD* will be dependent on the detector phase. Thus, provided that the two different types of clusters *CD*_1_ and *CD*_2_ have different excited-state lifetimes, then a phase dependent *CD* could in principle be detectable.

Inhomogeneous lifetime image (Gaussian or Lorentzian distribution). We will now consider a distribution of lifetimes. To do this we will use a numerical simulation to a particular distribution function with binning. The general form of such a probability distribution approach for the (unmodulated) cluster density is

(14) $${CD}_{tot}=\frac{{(\sum_{low}^{high}B_{n}{CD}_{n})}^{2}}{(\sum_{low}^{high}{B_{n}}^{2}{CD}_{n})}$$

where low and high refer to the shortest and longest lifetimes in the distribution, *B_n_* is the relative brightness of the nth state (taken to be the brightness of the longest lifetime state as 1 multiplied by the ratio of the lifetimes of state n and the longest lifetime state), and *CD_n_* is the probability of being in lifetime state n, multiplied by the total density of clusters, *CD_total_*.

The apparent total cluster density as a function of detector phase for the distribution case is given by the following expression:

(15) $${CD\left(\varphi_{D}\right)}_{tot}=\frac{{\left(\sum_{low}^{high}I_{psfn}\left(\varphi_{D}\right){CD}_{n}\right)}^{2}}{\left(\sum_{low}^{high}{CD}_{n}({I_{psfn}\left(\varphi_{D}\right))}^{2}\right)}\frac{{=(\sum_{low}^{high}\left({CD}_{n}(B_{n}+B_{n}m_{n}\cos\left(\varphi_{D}-\varphi_{n})\right)\right))}^{2}}{\left(\sum_{low}^{high}{CD}_{n}(B_{n}+B_{n}m_{n}\cos\left(\varphi_{D}-\varphi_{n}\right){)}^{2}\right)}$$

To constrain the nature of the distribution in the sum in Equation (15), it is prudent to assume a mathematical form linking all of the parameters. For the distribution models, we will stipulate that the fluorescent species are distributed as monomers and that the different environments encountered by the fluorophore result in differences in lifetimes and quantum yields.

We will first fix the total number of clusters at *CD_tot_*.

We will then denote the cluster density of particles with lifetime *τ_n_* to be *CD_n_*(*τ_n_*) = *P*(*τ_n_*)*CD_tot_*, where *P*(*τ_n_*) is the probability of finding a particle with a lifetime *τ_n_* (dependent on the parameters in the lifetime distribution).

The brightness of the nth particle, *B_n_* = *B_ref_* (*τ_n_*/*τ_ref_*), is scaled with the lifetime of the particle; this assumes that the particle is monomeric and the there is no change to the extinction coefficient of the particle with changes in environment, so the change in brightness is due to the change in the quantum yield only.

The modulation (*m_n_*) and the phase (*φ_n_*) are related to the lifetime *τ_n_* via Equations (3) and (4).

With these specifications, the form of the apparent cluster density as a function of the detector phase will depend only on the lifetime probability distribution *P*(*τ_n_*).

We will consider two different lifetime probability distributions.

The Gaussian lifetime distribution depends on the mean lifetime (*τ_mean_*) and the variance of the lifetime (sigma-squared) and is given by the following expression:

(16) $${P(\tau )}_{Gaussian}=Ne^{-\frac{{\left(\tau_{n}-\tau_{mean}\right)}^{2}}{\sigma^{2}}}$$

The Lorentzian distribution depends on a mean lifetime (*τ_mean_*) and a width parameter called gamma:

(17) $${P(\tau )}_{Lorentzian}=\frac{1}{\pi }\frac{\gamma }{({\tau_{n}-\tau_{mean})}^{2}+\gamma^{2}}$$

## 3. Results and Discussion

The goal of phase-sensitive fluorescence image correlation spectroscopy is to quantify the distribution of fluorescence entities in an image in terms of cluster density and fluorescence lifetime. To this end, we have simulated different lifetime-cluster distributions using the equations described in the Theory Section.

**Figure 1:**
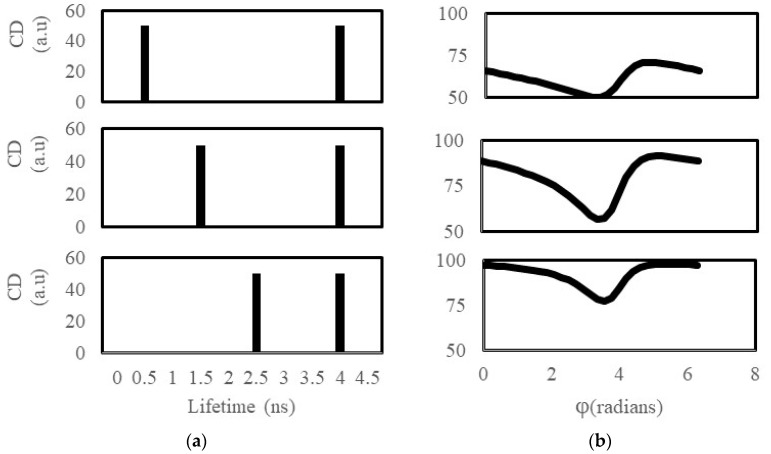
Phase-sensitive image correlation simulations for two-component lifetime distributions. (**a**) Lifetime-cluster density distributions. (**b**) Cluster density as a function of detector phase.

Figure 1 depicts three idealized distributions consisting of an equal density (i.e., CD_1_ = 50 and CD_2_ = 50) of two distinct lifetime states (see left panels in Figure 1). The two distinct lifetime states were 4 ns (common to all the distributions) and either 0.5, 1.5, or 2.5 ns, respectively. In the right-hand panels of Figure 1, we have computed the cluster density as a function of detected phase angle for the three different lifetime distributions. Focusing first on the 0.5 ns and 4 ns distribution (Figure 1, top panels), we can see in the phase-sensitive CD “spectrum” a clear minimum between 3 and 4 radians with a CD_min_ = 50, and a maximum between 4 and 6 radians with a CD_max_ close to 75. The minimum in this case is due to complete suppression of the 0.5 ns species, which is due to the detector phase being 180 degrees out-of-phase with the 0.5 ns species. The large difference in lifetime between the 0.5 ns and 4 ns species means that the complete suppression of one is possible without inadvertently suppressing the other. Note that the CD never reaches 100 for the 0.5 ns and 4 ns case because there is no detector phase angle that would make both species have the same brightness. Again, this is because of the already large difference in the lifetimes and hence the quantum yields of the two particles. Examining the results of the simulations with the 1.5 ns/4 ns distribution and the 2.5 ns/4 ns distribution reveals qualitatively similar shaped phase-sensitive CD spectra, however, the minimum and maximum CDs are clearly different and distinguishable. As the short lifetime approaches the longer lifetime, the minimum CD_min_ increases, indicating only partial suppression of the short lifetime states, and the maximum CD_max_ increases and approaches the total cluster density of the two combined species. The latter occurs because phase modulation of the brightness is sufficient to bring both particles within the same brightness.

**Figure 2:**
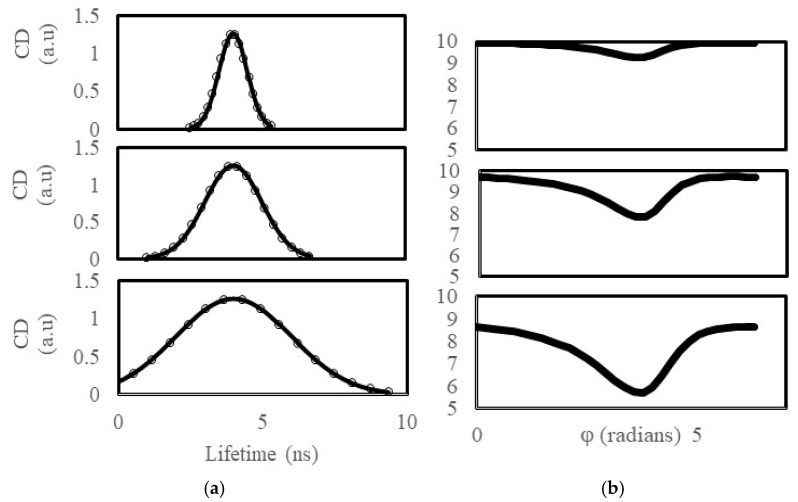
Phase-sensitive image correlation simulations for Gaussian lifetime distributions. (**a**) Lifetime distributions. (**b**) Cluster density as a function of detector phase.

Figure 2 represents simulations carried out on a Gaussian distribution of lifetime states with a total particle density of 10 a.u. The top, middle, and lowest panels represent the lifetime distributions with a common center lifetime of 4 ns and standard deviations increasing from 0.5 ns, 1.0 ns and 2.0 ns. The rightmost panels of Figure 2 represent the phase-sensitive CD spectra corresponding to the different lifetime distributions depicted on the leftmost panels. For the 4 ns center, 0.5 ns standard deviation model, the phase-sensitive CD spectrum has a CD close to 10 a.u. at all phase angles, except a slight dip down to CD_min_ = 9.3 a.u at phase angle = 3.8 radians. Increasing the heterogeneity in the lifetime distribution to a standard deviation = 1.0 ns, decreases the dip in the phase-sensitive CD spectrum to CD_min_ = 7.8 a.u. at phase angle = 3.8 radians. For the lifetime distribution with the greatest heterogeneity (s.d. = 2.0 ns), the phase-sensitive CD spectrum dips down to CD_min_ = 5.7 at phase angle = 3.8 radians. Qualitatively at least, the relative deviation in the maximum and minimum amplitudes of the phase-sensitive CD spectrum appears to be related to the extent of lifetime heterogeneity (as measured by the standard deviation in the lifetime distribution).

**Figure 3:**
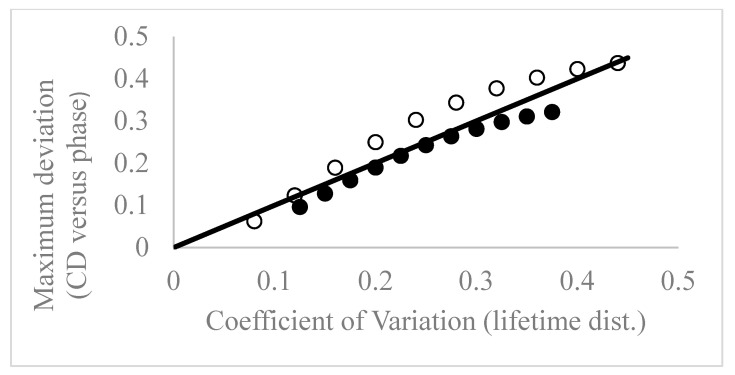
Phase-sensitive image correlation spectroscopy is sensitive to the width of the lifetime distribution. The vertical axis is the relative deviation in phase-sensitive cluster density and the horizontal axis is the co-efficient of variation in the Lorentzian lifetime distribution (empty circles (central lifetime = 2.5 ns; filled circles; central lifetime = 4 ns). Simulations were generated using Equations (17) and (15). Solid line is a plot of y = x and to guide the eye only.

To investigate the link between the degree of heterogeneity in the lifetime distribution and the phase-sensitive CD spectrum we carried out simulations for the Lorentzian distribution across a broader range of lifetime distribution parameter space. A summary of the results obtained is in Figure 3 is for two series of distributions: one series with fixed central lifetime of 4 ns and variable width (filled circles), and the other series with the central lifetime fixed at 2.5 ns and variable width (empty circles). In Figure 3, we have plotted the maximum (relative) deviation in the phase-sensitive CD spectrum (CD (max)-CD (min)/CD (max) as a function of the coefficient of variation in the lifetime distribution. From inspection of Figure 3, it is apparent that the relative deviation in the phase-sensitive CD spectrum is monotonically related to the relative width of the lifetime distribution. However, the relationship is not strictly 1:1, as can be seen by comparison with the linear solid line in Figure 3. Data from the 4 ns simulation fall slightly below the line, while data from the 2.5 ns simulations fall slightly above the line.

While the two component and lifetime distribution models both generate phase-dependent cluster densities, it is of interest to ask whether these distributions are distinct. That is, can two lifetime images that have the same average lifetime produce distinct phase-sensitive CD spectra? To better define this question, we have simulated two component and Gaussian lifetime distributed systems where the average lifetimes are constrained to be identical. For this purpose, we calculated the so-called fluorescence-weighted average of the phasor components, i.e., G = mcosφ and S = msinφ for a given two-state model, and then optimized the mean and standard deviation of the Gaussian lifetime distribution so that the corresponding G and S values were within 0.001 of the corresponding two-state model values. Table 1 collects some examples of iso–phasor distributions with pertinent lifetime distribution parameters and key features of the phase-sensitive CD spectra. In the first row, we have simulated a two-component model with 35 clusters/area (CD_1_) with lifetime 4.2 ns, and 15 clusters/area (CD_2_) with lifetime 0.24 ns. The phase-dependent CD spectrum has a minimum CD reflecting the 4.2 ns clusters with CD_min_ = CD_1_ = 35, which increases to CD_max_ = 38. The Gaussian lifetime distribution with identical phasor components with lifetime center of 4 ns and width 0.86, and the identical total number of clusters of 50 has a phase-dependent CD spectrum, which is distinct from the two-component model. The Gaussian distribution has a CD_min_ of 41 and a CD_max_ of 49, which is different from the CD_max_ and CD_min_ values from the two-state model by >20% and >15%, respectively. The relative deviation in the phase-sensitive CD spectra for the two-state model is 3/38, compared with 8/49 for the Gaussian model.

Another simulation with two-components (CD_1_ = 40, tau_1_ = 2.8 ns; CD_2_ = 20, tau_2_ = 0.6 ns) generated phase-dependent CD spectrum with CD_max_ = 52 and CD_min_ = CD_1_ = 40, and a maximum relative deviation of 8/52 = 0.15. The iso-phasor Gaussian distribution (total number of clusters = 60, lifetime center = 1.7 ns, and lifetime width = 1.3 ns) showed larger differences in CD_max_ and CD_min_ from the phase-sensitive CD spectra (CD_max_ = 40; CD_min_ = 20) amounting to a maximum relative deviation of 20/40 = 0.5. These two examples serve to demonstrate that in certain situations, image correlation spectroscopy analysis of phase-sensitive fluorescence images may enable improved discrimination between different types of lifetime and cluster distributions that cannot be discerned via phasor analysis alone. We will next provide an example of such an analysis workflow using real data acquired with our full-field homo-dyne frequency-domain microscope.

To demonstrate one application of our analysis approach, we will refer to data collected as part of a previous study aimed at looking at the dynamics of Golgi membranes in the interior of cervical cancer cells [16]. In that research, we labelled the Golgi membranes with a Golgi-specific dye (NBD-C_6_-ceramide) and recorded FLIM images at different wavelengths. The wavelength dependence of the lifetimes was analyzed to extract an average lifetime for population decay of the excited state and a relaxation time attributed to membrane dynamics. Data acquired at the bluest wavelength (530 nm) were dominated by the population decay processes, while the spectral relaxation processes were revealed at the longer wavelengths (600 nm).

Here, we will present data collected in the 530 nm wavelength region to focus on the inherent heterogeneity in the population decay processes, which, for NBD-membrane probes, are sensitive to the order of the membrane [17]. The intent here is to illustrate how we analyzed the data rather than to provide an exhaustive study on membrane probe properties.

**Figure 4:**
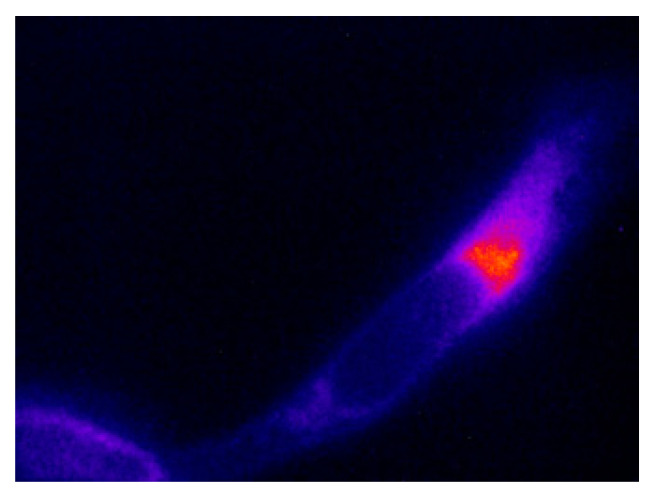
Fluorescence image of a cervical cancer cell stained with NBD-C_6_-ceramide probe. The red-orange region denotes the Golgi membrane region, while the blue regions denote the outer (plasma) membranes regions of the cell.

Figure 4 contains a fluorescence image of NBD-C_6_-ceramide in a HeLa cervical cancer cell acquired with our full-field FLIM microscope (excitation:474 nm, emission:530 nm). The most intense staining occurs at the Golgi membranes (red region of the cell image in Figure 4), while the outer membranes are stained less significantly (blue region of the cell image in Figure 4).

**Figure 5:**
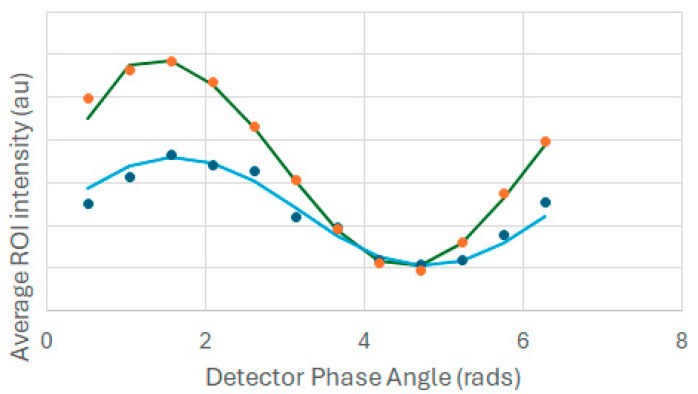
Phase-sensitive average intensities from rhodamine 6G solution (orange symbols) and from a region of external membrane of a cervical cancer cell stained with the NBD-C_6_-ceramide probe (blue symbols). Solid lines denote fits to Equation (5), using sum-of-least-squares minimization.

Figure 5 depicts phase-sensitive fluorescence from a region of interest near the outer membrane of the cell, and phase-sensitive fluorescence from a solution of a lifetime standard (rhodamine 6G, lifetime = 4.1 ns). The data points refer to the average intensity as a function of detector phase and the solid lines are fits to Equation (5). As can be seen in Figure 5, the fluorescence from the cell is delayed in-phase relative to the rhodamine 6G solution, which implies a longer than 4.1 ns lifetime of the membrane probe excited state. After correcting for instrumental phase lag and demodulation (using the rhodamine 6G solution), the average lifetime from the phase-sensitive average intensity in the outer membrane region was close to 7 ns.

**Figure 6:**
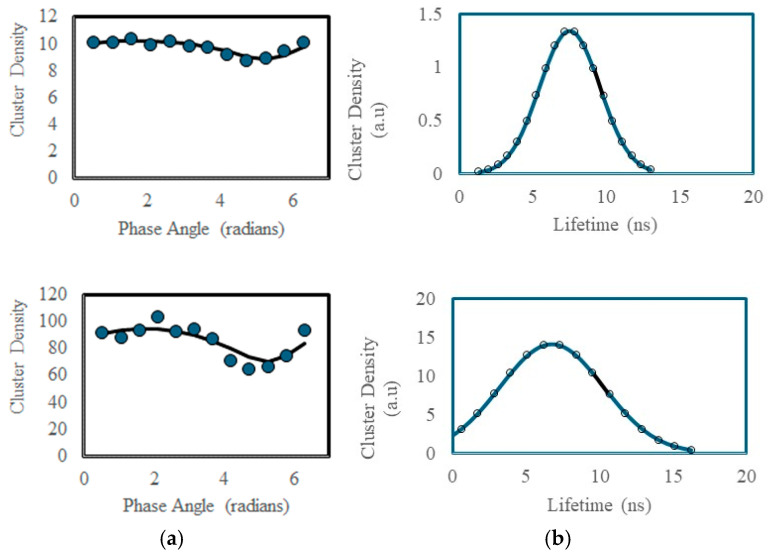
Phase-sensitive image correlation spectroscopy applied to a membrane probe in a cervical cancer cell. (**a**) Phase-sensitive cluster densities from outer membrane (**upper**) and Golgi membrane (**lower** panel); Solid lines are fits to Equations (15) and (16) using sum-of-least-squares minimization. (**b**) Calculated lifetime histograms from outer membrane (**upper**) and Golgi membrane (**lower**).

Image correlation analysis on the phase-sensitive images delivered cluster densities (units: number per beam area) as a function of detector phase. Figure 6 contains the CD as a function of phase trajectories from an outer-membrane region (upper plot) and from Golgi region (lower plot). It is notable that the density of probes is higher in the Golgi region (CD = 100/BA) than the outer membrane (CD = 10/BA), and the relative deviation is larger in the Golgi region than in the outer membrane region. To interpret the phase-sensitive CD spectra, we fit the data to Gaussian lifetime distribution (Equations (15) and (16) with instrumental demodulation and phase delay included). The solid lines in Figure 6a denote the fits to the CD versus phase plots, while the lifetime histograms are shown in Figure 6b. Of note is that the lifetime distribution in the Golgi membrane is broader (lifetime width = 3.5 ns) than in the outer membrane (lifetime width = 2.1 ns), despite the stronger staining by the membrane probe in the Golgi region.

To estimate the uncertainties in the derived lifetime distribution widths, we re-sampled the data (with replacement) and re-fit the data. The derived lifetime widths were 3.6 ± 0.2 ns for the Golgi region and 2.1 ± 0.1 ns from the plasma membrane region. According to our analysis in both regions of the membrane, the probes are at high density and therefore contain many more molecules in each resolvable element of the microscope. We therefore calculated the effect of molecular averaging on lifetime distribution widths, assuming about 10 molecules per average in the plasma membrane and 100 molecules per average in the Golgi membrane. The calculations revealed that the predicted lifetime width from pre-averaging in the Golgi membrane to be 0.22 ns and 0.57 ns for the plasma membrane. These values can be compared with measured modulation lifetime distribution widths of 0.29 ns and 0.66 ns from regions of interest at the Golgi and plasma membrane, respectively). This analysis underscores the importance of pre-averaging on detected distribution parameters from lifetime images.

According to Herrmann’s lab [17], an NBD-membrane-probe lifetime of about 7 ns is consistent with a liquid-disordered phase, while a lifetime close to 11 ns is associated with a liquid-ordered phase. Since both lifetime histograms are centered closer to 7 ns than 11 ns, it is suggested that perhaps the NBD-ceramide probe is targeted to largely liquid-disordered regions of the membrane. However, the broad lifetime distributions derived here suggest that the NBD-C_6_-ceramide probe may be in a wide range of environments. Other researchers have proposed that NBD membrane probes locate in different transverse regions of the bilayer, in regions differing in polarity and hydration [18]. If lifetime heterogeneity was due to lateral heterogeneity of probes dispersed in different membrane domains, then a STICs-type approach [13] to different phase-sensitive images would reveal a loss of cross-correlation. On the other hand, if lifetime heterogeneity was due to transverse heterogeneity (perpendicular to the membrane plane), only then should good cross-correlation still be observed between different fluorescence images acquired at different phase settings of the detector. More work is needed to fully interpret the results, which would benefit from studies on pure membranes (dipalmitoylphosphatidylcholine DPPC for the liquid-ordered phase and dioleoylphosphocholine DOPC for the liquid-disordered phase).

To the best of our knowledge, this is the first application of image correlation spectroscopy to phase-sensitive fluorescence images and represents a novel approach to measuring lifetime heterogeneity in an image, i.e., by linking the density of fluorescent particles (i.e., molecules) with excited-state lifetime. The approach is anticipated to be of maximum utility in images where fluorescence intensity and fluorescence lifetime appear spatially diffuse, and when spatial averaging perturbs the true lifetime distribution. However, there are important limitations of the method devised here. First, we assumed that differences in the detected cluster density with the detector phase are due to lifetime heterogeneity only. Other sources of changes in cluster density were not considered in the simple model developed here. For example, changes in apparent cluster density could occur due to aggregation or movement of fluorescent species. Analysis of unmodulated images using temporal image correlation spectroscopy could be utilized to ascertain particle motions, and conventional ICS could be used to assess changes to the cluster density due to particle movement. In conventional FLIM, particle motion causes a broadening of the lifetime distribution and can completely distort even the average lifetime values because of intensity fluctuations at the single pixel level [5]. Provided that the same number of particles is sampled in psf–ICS, in principle, the correct determination of average lifetime and lifetime heterogeneity is possible even with particle motion. This is because only the average intensity and average density in a much larger region of interest spanning thousands of pixels would be determined, and so it would be less sensitive to fluctuations. Second, psf–ICS is limited by the magnitude of the lifetime heterogeneity in the image and the precision of the ICS analysis. Our simulations indicate that for a Lorentzian lifetime distribution, a coefficient of variation of about 5% is probably the smallest degree of heterogeneity that could be detected. The reader is referred to an excellent paper on ICS simulations that shows how image size and particle density influence the accuracy and range of ICS measurements [11]. Third, we have used simple models of lifetime heterogeneity including two-state, Gaussian, and Lorentzian distributions. These distributions are easiest to fit to data because they contain only a few parameters. In principle, other distributions could be considered. As we learn more about what affects lifetime values and as computer simulations approach that of the living cell, it may be possible to calculate the distribution of lifetimes in a cell and then compare this with the psf–ICS. Fourth, experimental implementation of psf–ICS requires access to light sources and detectors that can be modulated at high frequencies in the tens of MHz range. For convenience we utilized a commercial wide-field frequency-domain system mounted on a research grade fluorescence microscope, and this is probably the easiest route to implementation for the non-instrumentalist. Our system uses a modulated LED as the excitation source and an intensifier-CCD combination as the modulated detector. In principle, any instrument will be fine if fluorescence images can be generated and the images can be collected at different relative phases of the fluorescence emission and the detector.

It is useful to compare our approach to other approaches in the literature. In our previous work, we have used the phasor plot in conjunction with image correlation spectroscopy to determine the cluster densities and relative brightness of species in the context of a two-component lifetime system [15]. The phasor approach provides a means to visualize the distribution of lifetimes presented as a two-dimensional plot [19,20,21] and in its current State of the Art can resolve up to three to four discrete lifetime components in the image of a cell [22]. The unmixing of those lifetime components and the use of ICS could yield cluster densities and sizes of different discrete lifetime species. However, such an approach is limited to a finite number of lifetime species and would not work for a continuous distribution of closely-spaced lifetimes. Another approach is single molecule FLIM [23]. This represents the ultimate resolution of FLIM measurements, allowing true lifetime distributions to be built up from single particle measurements [23]. The psf–ICS method has the advantage of high sampling efficiency and can be applied when there is more than one fluorophore per resolvable element of the microscope.

The paper here represents the first step in psf–ICS. Cross-correlation analysis [11] could be applied between images collected at different detector phases to reveal the extent to which spatial fluctuations and lifetime differences are correlated. For example, if there was a spatial gradient of lifetimes in an image, cross-correlation between different detector phases would reveal a cross-correlation image whose maximum would shift in position. Alternatively, if the particles with different lifetimes were dispersed randomly, then the cross-correlation image maximum would remain in the same position, but the peak magnitude would decrease as a function of detector phase. In principle, this analysis could be extended further by incorporating particle motion. In this case, cross-correlation between different phases at different times could reveal diffusion. The diffusion would be evidenced by a cross-correlation that decreases with increasing lag time, while the lifetime heterogeneity would be evidenced by psf–ICS (correcting for diffusion-influenced particle fluctuations).

The psf–ICS method and analysis can be performed on images where there are fluorescence objects dispersed at different densities and with different fluorescence lifetimes. We envisage that the method will be particularly useful for fluorescence probes that sense subtle differences in hydration, polarity, pH, or refractive index. In the cellular milieu, these properties change dramatically in different (sub) cellular compartments and at interfaces of membranes and biological macromolecules, and may be a function of cellular state or polarity (i.e., during cell division, cell growth, cell migration, or cell death). Coupled with appropriate excitation formats (total internal reflection or light-sheet), psf–ICS could potentially be adapted to look at either surface-selective phenomena or used to interrogate the inner workings of a model organism.

## 4. Materials and Methods

Phase-sensitive fluorescence images were collected using a commercial FLIM instrument as described previously [13]. Using the LIFA-FLIM 2.1 software from Lambert Instruments(Gronigen, The Netherlands), the reference stack and sample stack were imported as *.ids format images and then opened in the ImageJ (Fiji) open-source microscopy software program [24] Each image stack contained 12 phase-sensitive images. Regions of interest of sizes 32 × 32 or 64 × 64 pixels were selected and cropped. Average image intensities were calculated using the measure function in ImageJ. Autocorrelation analysis was performed using the correlation option in FD Math; the resulting images were corrected by normalizing for the number of pixels and the square of the average image intensity, and finally subtracting one. The inverse of the autocorrelation at zero lag was calculated as the cluster density or CD in units of number per beam area.

## Figures and Tables

**Table 1:** Phase-sensitive CD spectra can distinguish different lifetime distributions with identical average phasors. Note that phasor components are unitless and the CD values are in units of number per beam area.

Phasor	Lifetime Model	CD_Max, Min_
(0.486, 0.489)	35(4.2 ns), 15(0.24 ns)	38, 35
(0.486, 0.489)	center(4 ns), width(0.86 ns)	49, 41
(0.698, 0.439)	40(2.8 ns), 20(0.6 ns)	52, 40
(0.698, 0.439)	center(1.73 ns), width(1.31 ns)	40, 20

## Data Availability

Data are available on request to the author.
